# Effects of restricting movement between root and canopy populations of woolly apple aphid

**DOI:** 10.1371/journal.pone.0216424

**Published:** 2019-05-06

**Authors:** Robert J. Orpet, Vincent P. Jones, John P. Reganold, David W. Crowder

**Affiliations:** 1 Department of Entomology, Tree Fruit Research and Extension Center, Washington State University, Wenatchee, Washington, United States of America; 2 Department of Crop and Soil Sciences, Washington State University, Pullman, Washington, United States of America; 3 Department of Entomology, Washington State University, Pullman, Washington, United States of America; University of Manitoba, CANADA

## Abstract

Movement of insect pests between spatially subdivided populations can allow them to recolonize areas where local extinction has occurred, increasing pest persistence. Populations of woolly apple aphid (*Eriosoma lanigerum* [Hausmann]; Hemiptera: Aphididae), a worldwide pest of apple (*Malus domestica* [Borkhausen]), occur both below- and aboveground. These spatially subdivided subpopulations encounter different abiotic conditions, natural enemies, and control tactics. Restricting movement between them might be an effective management tactic to decrease woolly apple aphid persistence and abundance. We examined this possibility in the field, using sticky barriers to restrict upward woolly apple aphid movement to tree canopies, and in the greenhouse, using mulches and sand amendments to restrict downward movement to roots. In the field, blocking aphid movement up tree trunks did not decrease the number of colonies in tree canopies. Instead, sticky-banded apple trees had higher aphid colony counts late in the study. Earwigs, which are woolly apple aphid predators, were excluded from tree canopies by sticky bands. In the greenhouse, fewer root galls (indicative of aphid feeding) occurred on trees in sandy potting media and on those with mulch (wood chips or paper slurry). Our results suggest that upward movement is less important than other factors that affect aboveground aerial woolly apple aphid population dynamics. In addition, apple orchards planted in sandier soils or with mulches may be partially protected from woolly apple aphid root feeding.

## Introduction

Many pest species move into crops from habitats that provide spatial or temporal refuge from control tactics, such as neighboring fields or natural habitats [1,2]. Movement between such subdivided pest populations promotes pest stability (variation in abundance across time) and persistence (rates of local extinction) [3,4], perhaps confounding management efforts to reduce population density. Management strategies that control pests across subpopulations in synchrony should produce large reductions in pest density and may lead to regional extinction [5]. However, it is not always possible to manage pests before they disperse from source locations. In these situations, a possible alternative tactic would be to restrict pest movement with physical barriers [1].

For pests that move into and out of the soil, restricting movement might reduce their abundance in both environments. This may be an effective tactic, as underground (edaphic) pest populations are often difficult to manage and can be a source of aboveground (aerial) populations [6]. Barriers applied to soil, such as mulches and row covers, can indirectly effect edaphic pest populations by modifying natural enemy communities, animal orientation behavior, and soil temperature [7,8]. However, research on such barriers has not focused on pest movement through soil. Likewise, soil qualities can influence edaphic pest abundance [9,10], but evidence on whether this affects conspecific aerial populations is lacking. Most research linking edaphic and aerial herbivores has focused on indirect plant-mediated interactions, not movement [11,12].

Woolly apple aphid (*Eriosoma lanigerum* [Hausmann]; Hemiptera: Aphididae) is a worldwide apple (*Malus domestica* [Borkhausen]) pest with nymphs (‘crawlers’) that can move into and out of the soil year-round, although movement is reduced during winter [13–17]. Aerial and edaphic woolly apple aphid populations are economically damaging and difficult to manage [18–21]. Resistant rootstocks are not widely used [22,23], and belowground natural enemies are generally ineffective or have unknown effects on suppression [24–27]. Although certain systemic insecticides are effective against edaphic colonies, they are not available for use in organic orchards [23,28–30]. It has been speculated that edaphic woolly apple aphid populations contribute to aerial population growth and persistence [14–17], so restricting aphid movement into and out of the soil with mulch or other barriers may be a reasonable control tactic.

Here we addressed the relationship between edaphic and aerial populations of woolly apple aphid and the management potential of physical barriers. In the field, we tested whether blocking crawler movement with barriers affected numbers of aerial aphid colonies. Several past studies used sticky barriers to monitor upward and downward woolly apple aphid crawler movement [13–17], but most could have been confounded by lateral aphid movement between treated and untreated trees. Our experiment therefore consisted of spatially separate sections of trees that all either received or did not receive sticky barriers. We also tested whether mulches and sand content of potting media affected woolly apple aphid colonization of greenhouse apple tree roots.

## Materials and methods

### Field experiment

#### Location and design

**Fig 1:**
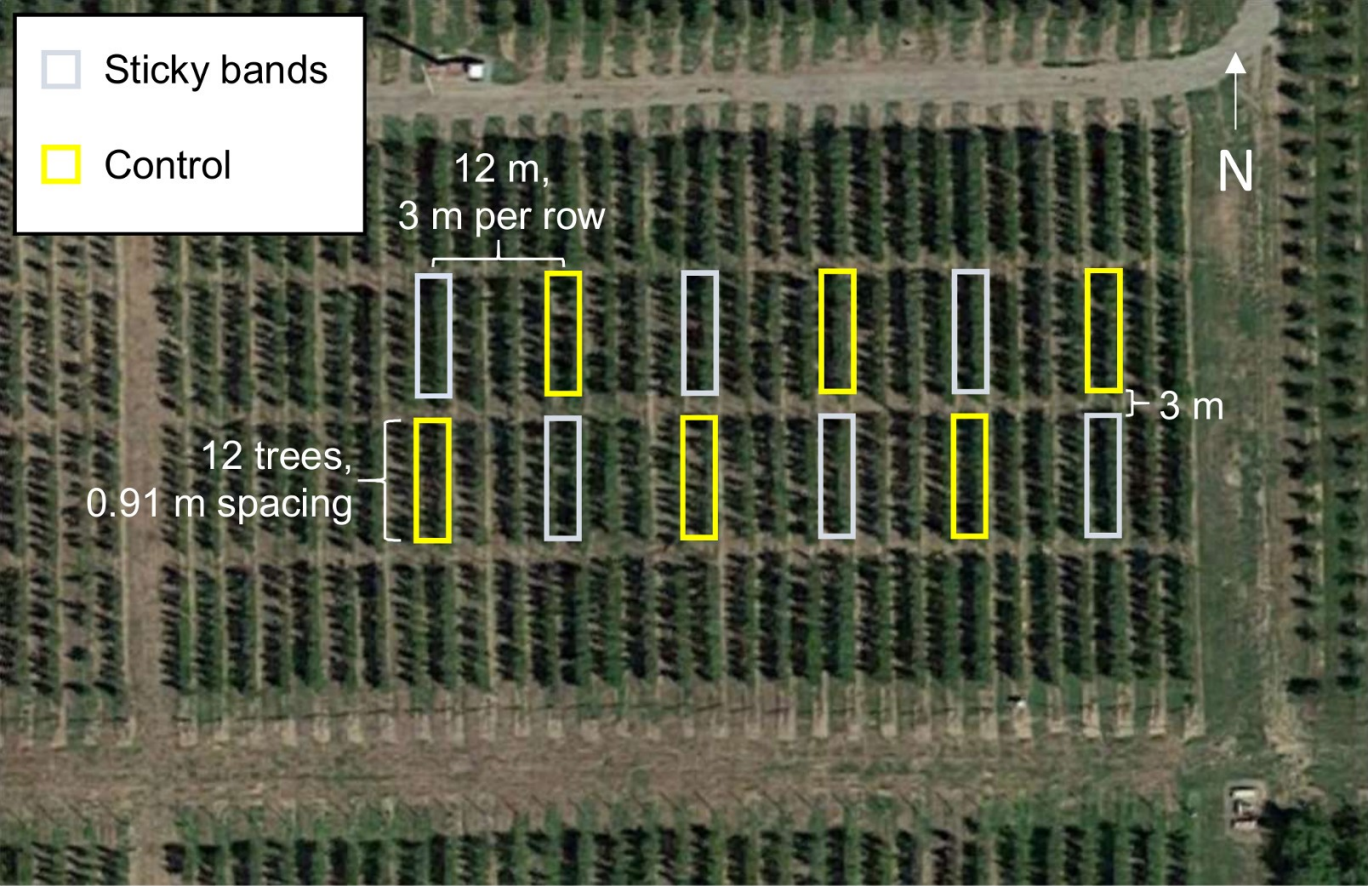
Diagram of the study area showing the locations and spacings of sticky band and control section replicates.

In 2017, we assessed aerial woolly apple aphid population dynamics in the presence and absence of edaphic-originating crawlers in the field. The experiment took place on a 0.5 ha plot of apple trees planted in 2007 at the Washington State University Sunrise Research Orchard (47.31°N, 120.07°W) near Rock Island, WA (Fig 1). At the time of our study, trees were between 3 to 4 m in height. Each of the 31 rows in the plot had four sections of 12 trees, with 3 m of open ground between sections. Trees were spaced 0.91 m apart and tree rows were spaced 3 m apart. Trees in each row were from a single cultivar, alternating between Fuji, Golden, Gala, and Jonagold across rows. All were on M9 rootstock, which is considered very susceptible to woolly apple aphid [22], and had similar canopy sizes. We selected two interior sections of six Fuji rows for this study. In each row, one of these two sections received sticky bands around the base of each tree’s trunk (sticky band treatment), and the other section was not manipulated (control). Treatment assignments alternated between the more northern vs. more southern study section between adjacent study rows (Fig 1). The east-most sticky-banded section contained only 11 trees as one had died in a previous year, so our study included 143 total trees (71 sticky-banded and 72 control). The plot received minimal management inputs and no insecticides.

#### Sticky bands and crawler monitoring

Tanglefoot (The Tanglefoot Company, Grand Rapids, MI) bands were applied to all sticky band treatment trees between 8 July and 10 July, 2017. Bands were 10 cm high × 0.6 cm thick, applied onto layers of cloth tape (North American Tapes, Watertown, NY) 20 cm aboveground. The tape was wrapped tightly on a smooth trunk section, with no space for aphids to crawl under. Once a month, barriers were refreshed by rubbing them with fresh Tanglefoot. To prevent lateral crawler movement into sticky band treatment sections from other sections, trellising wires were coated with a 4 cm wide × 0.6 cm thick layer of Tanglefoot, and any plant material beyond this barrier was pruned. Suckers and water sprouts were removed from all study trees with shears.

We did not monitor edaphic populations because current methods, such as uprooting trees [21,28,31] or taking soil cores [29], are destructive and cannot be used repeatedly on the same tree. Therefore, to confirm that edaphic populations were present and aphids were attempting to move upward during out experiment, we wrapped a 1.9 cm wide band of double-sided tape (Scotch Brand, 3M Company, St. Paul, MN) directly below each Tanglefoot barrier to monitor upward crawler movement. Starting on 11 Jul and until 31 Oct, the tapes were collected and replaced weekly, except for the final collection on 31 Oct, where tapes had been deployed for two weeks. All woolly apple aphids found on tapes were counted in the laboratory using a stereoscope. We made no attempt to quantify downward-moving aphids (which would be blocked by the Tanglefoot) as past studies suggest they are not correlated to aerial population declines [15–17].

#### Aerial insect monitoring

We counted the number of woolly apple aphid colonies on the west side of all study trees up to a height of 2 m (corresponding to about one fourth of the total tree area) typically once a week from 5 July to 31 Oct. Each infested leaf axil and infested wound on bark (e.g., pruning cuts) was considered an individual colony. A previous study [32] found similar seasonal trends in woolly apple aphid abundance both below and above 2 m height on apple trees, suggesting our sampling strategy was suitable for monitoring aphid population dynamics. The decision to inspect only the west side of trees allowed us to avoid traveling between the east and west side of the dense tree rows, which contained trellising wires, and prevent damage to trees. We assumed that woolly apple aphid population dynamics would be similar on the east and west side of trees, so we arbitrarily selected the west side for our observations prior to the start of the study.

Occurrence of winged woolly apple aphids was assessed one to two times per week by collecting up to 20 randomly selected woolly apple colonies from shoots. These collections were made during haphazard walking outside of the study sections but within the 0.5 ha study block, starting on 3 July and ending on 7 November. All colonies from a collection date were pooled and washed over filter paper, and then the number of woolly apple aphid nymphs, nymphs with wing buds, adults, and adults with wings, were counted under a dissecting stereoscope.

To monitor European earwig (*Forficula auricularia* L.; Dermaptera: Forficulidae), an aphid predator that may be excluded from tree canopies by sticky bands [33], each study section received five earwig shelters, one on every other tree starting with the second tree. Shelters consisted of rolled strips of single-face corrugated cardboard (Uline, Pleasant Prairie, WI), approximately 7.5 × 35.5 cm. Earwigs are nocturnal and readily hide in such shelters during the day [33]. Each rolled strip was affixed to a tree using a rubber band, in contact with trunk and ≈1 m from the ground. Earwigs were shaken out of all shelters, counted, and released on the orchard floor on ca. weekly visits from 18 July to 31 Oct. Sometimes shelters fell or were missing, resulting in a mean number of shelters counted per section per visit of 4.3 (SD = 0.9).

#### Field experiment statistical analysis

We used repeated measures generalized linear models to assess the effect of sticky bands on the abundance of aerial woolly apple aphids and earwigs. Separate models were constructed with either woolly apple aphid colony counts (pooled for all trees in a section per each visit) or earwig shelter counts (pooled for all shelters in a section per each visit) as response variables. Study sections were treated as replicates (*N* = 6 per treatment). The explanatory variables were experimental treatment (sticky band or control), time, and the time × treatment interaction. Time was treated as a categorical variable as insect counts varied non-linearly with respect to time. Study section was included as a random effect in models to account for the repeated measures structure. Based on the distribution of the response variables, each model was fit with a negative binomial response distribution (with ‘NB1’ parameterization) using the ‘glmmTMB’ package [34] in R [35]. Log_*e*_ ‘sampling effort’ (i.e., the number of trees or number of earwig shelters in a section) was included as an ‘offset’ to account for the one dead tree in a sticky banded row, and for when earwig shelters were missing. We observed declining numbers of earwigs starting in October, when earwigs transition to underground nests for the winter [36] so we excluded the final observation day, when no earwigs were found, from earwig analysis.

We also calculated cumulative insect-days, which represent the amount and duration of insect occurrence (either woolly apple aphid colonies or earwigs) in one expression as:

$$\sum \left(D_{i+1}-D_{i})[\left(Y_{i}+Y_{i+1}\right)/2\right]$$

where *D*_*i*_ and *D*_*i*+1_ are adjacent observation time points (days) and *Y*_*i*_ and *Y*_*i*+1_ are insect counts on those days [37]. Insect-days were calculated from 13 July (the first observation after Tanglefoot bands were applied) until the end of the study on 31 October. We used a two-sample, two-tailed *t*-test to determine whether cumulative insect-days differed for aphids and earwigs between the sticky band and control sections (*N* = 6 for each treatment).

All relevant data from our field experiment and winged aphid monitoring are available in supporting information (S1 and S2 Tables).

### Greenhouse experiment

#### Trees, mulches, and experimental design

We investigated the effects of two types of potting media and three mulches (including a no-mulch control) on aphid root colony and gall abundance in a factorial greenhouse study (2 potting media × 3 mulches × 12 replicates = 72 study trees, although one tree died during the study and was excluded from analysis). Apple trees (non-grafted M9 NIC29 selection rootstock liners, 0.95-cm diameter, from Willow Drive Nursery, Ephrata, WA) were potted in 22.2 cm diameter pots on 28 to 29 March 2016. The trees were potted with 3 L of either a ‘plain’ medium made from equal volumes of perlite, vermiculite, and sphagnum peat moss, or a ‘sandy’ medium of 67% sand content. The sandy medium was created by mixing 6 L of ‘multi-purpose’ sand (The Quikrete Companies, Atlanta, GA) with 3 L of plain medium per 9 L. Potted trees containing the different media were evenly interspersed over two greenhouse benches.

For mulch treatments, on 16 to 17 May 2016, pots were amended with one of three treatments: (1) nothing (control); (2) 10 cm depth of wood chips; or (3) wet paper slurry. Drainage holes in pots were covered with fine mesh to prevent aphid access to roots. Wood chips ranged in size from < 1 mm fibers to 15 cm long, 5 cm diameter chunks, and came from chipped trees of a commercial apple orchard. The paper slurry consisted of recycled paper pulp in water (Keyes Fiber, Wenatchee, WA), had a wet density of 1.07 kg per L and a dry density of 0.051 kg per wet L. To apply this treatment, 700 mL of wet slurry were first added to pots, and then on the following day an additional 100 mL mixed into 500 mL of water was added to seal cracks and augment thin sections. The dried slurry formed solid paper-like mats, approximately 4 to 6 mm thick, across the surface of the potting media. This mat remained solid during watering, but shrank when dry, pulling back up to 5 mm from tree trunks and sides of pots.

The potted trees were aerially infested to provide a source of downward-moving aphids to attempt root colonization. On 1 June and 9 June 2016, each study tree received one field-collected 4 to 6 cm long woolly apple aphid colony placed in its canopy. Trees with no apparent infestation on 17 June received another field-collected colony the same day.

#### Aphid and root dry weight quantification

**Fig 2:**
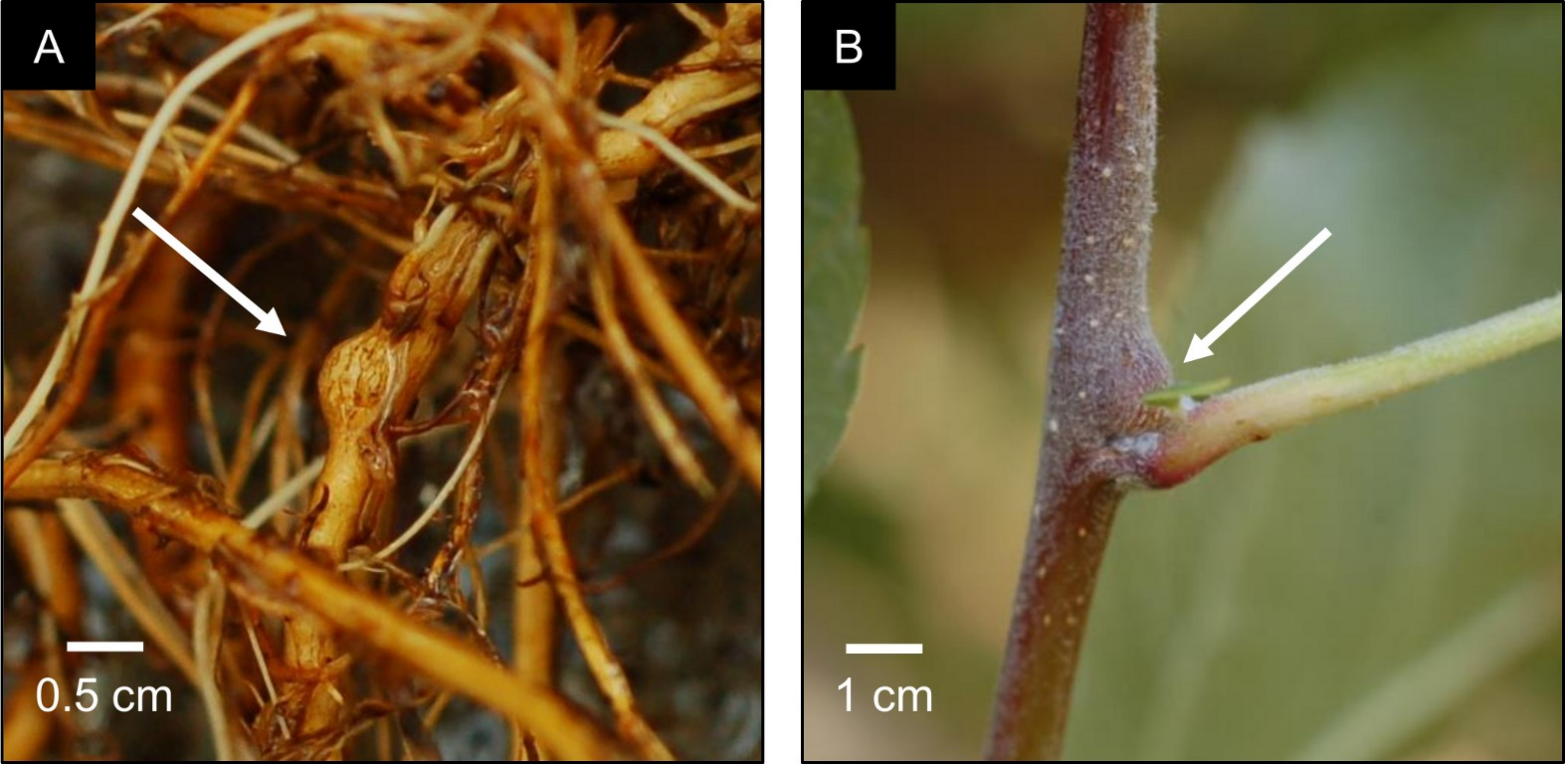
Woolly apple aphid induced galls. (A) A root gall observed on a greenhouse tree, and, for comparison, (B) an aerial gall formed on a tree in the field around a small woolly apple aphid colony.

On 29 July, the number of aerial colonies on leaf axil bases and woody tissue of each tree was counted. On the same day, root systems of all trees were removed from pots for inspection. Patches of visible aphids or wax fibers produced by aphids on roots were considered indicative of individual colonies when separated by more than 1 cm. After root colonies were counted, the roots were rinsed and scrubbed with a paint brush to remove potting media. Then, the number of galls were counted, which were easily identified as bead-like spherical swellings (Fig 2). Finally, root systems were dried at 55°C for 5 days and weighed to the nearest 0.1 mg.

#### Greenhouse experiment statistical analysis

We first used multiple regression models to assess effects of mulch treatment (control, chips, slurry), potting media (plain, sandy), and their interaction on four responses: (1) number of aerial aphid colonies, (2) root dry weights, (3) number of aphid root colonies, and (4) number of root galls. Linear models were appropriate for all response variables except for root gall counts, which were analyzed with a generalized linear model using a Poisson distribution and log link function. These analyses were conducted in R using the ‘lm’ or ‘glm’ functions (for the linear and generalized linear models, respectively). Significance of effects were assessed by *F*-tests for the linear models and *χ*^*2*^ deviance goodness of fit tests for the generalized linear model.

As the response variables above may have been correlated (i.e., they may have affected each other, in addition to being affected by the experimental treatments), we next used piecewise structural equation modeling to assess direct and indirect effects of the experimental treatments [38]. Our model set consisted of: (1) a linear model of root colonies distributed by potting media treatment, root dry weight, and aerial colonies, (2) a generalized linear model with a Poisson distribution and log link function of root galls distributed by potting media treatment, mulch treatment, root dry weight, and aerial colonies, and (3) a linear model of root dry weight distributed by potting media treatment. In a structural equation model, justifications for each variable and the directionality of effects are needed. Our model justifications were based on *a priori* predictions and the models described above. We hypothesized that (1) sandy media would reduce woolly apple aphid movement to roots, decreasing root colonies and galls, (2) lower root dry weight, as a proxy for root system size, could reduce the number of root woolly apple aphid colonies and galls because of less available space for colonies to form, (3) a greater number of aerial colonies would result in more root colonies and galls because more aphids would attempt downward movement to roots, (4) both mulch types would reduce the number of root galls, but not the number of root colonies (following results of multiple regression models), and (5) root dry weight would be affected by potting media (following results of multiple regression models). Damage from root colonies and root galls were not expected to decrease root mass over the short term of this study [19]. Our structural equation model was fit using the ‘piecewiseSEM’ package in R, in which the set of hypothesized relationships is considered consistent with the data when Fisher’s *C* statistic tested with a *χ*^2^ distribution generates a *P* value > 0.05 [38]. All relevant data from our greenhouse experiment are available in supporting information (S3 Table).

## Results

### Field experiment

**Fig 3:**
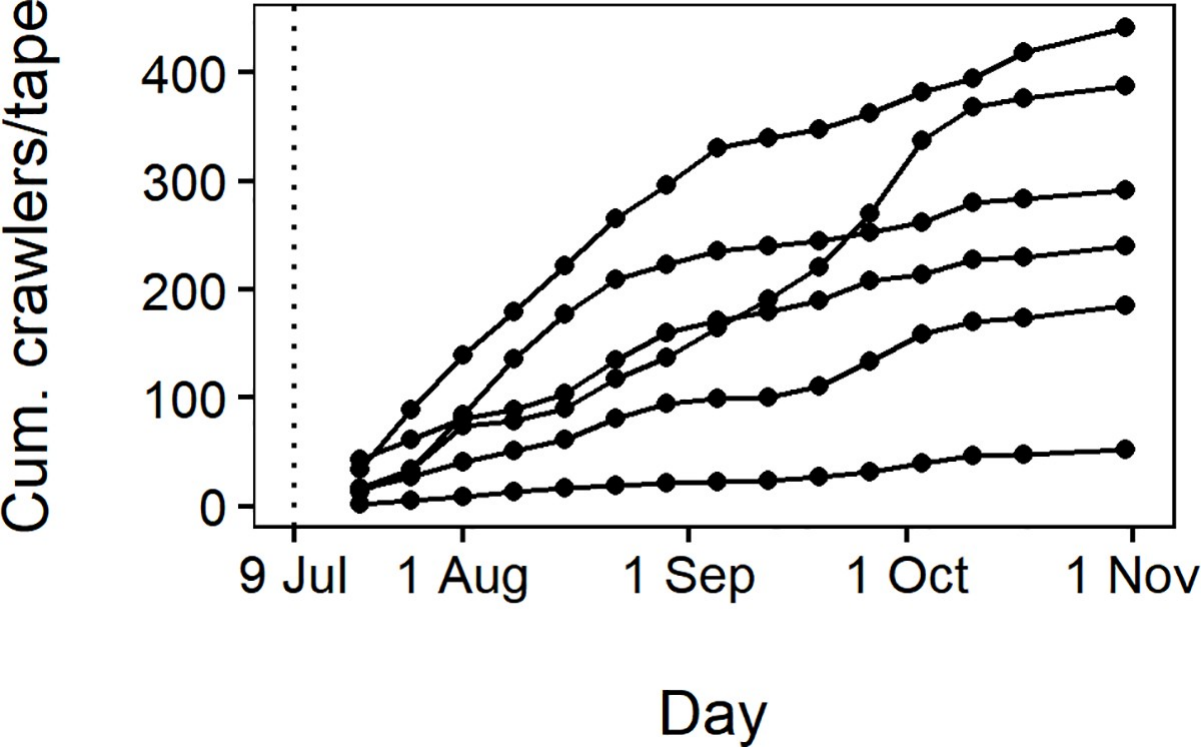
Cumulative upward-moving crawlers collected on double-sided tapes per tree. Each line represents one of the six sticky band sections. The vertical dotted line shows when sticky bands were applied.

Upward-moving crawlers were trapped on double-sided sticky tapes throughout the observation period. Among the six sticky band sections (replicates) (Fig 1), there was no clear seasonal peak in upward woolly apple aphid movement, but there was variation in the cumulative number of crawlers up to Nov 1 caught per tree, ranging from 53 to 442 (Fig 3). These numbers may underestimate the true number of upward-moving aphids, as some aphids might be deterred by the tapes and avoid attempting to walk over them. In addition, some aphids may have succeeded in walking over the tapes, as we often observed ants in the field walking over double-sided sticky tapes. However, ant trails, which were seen on tree trunks in control sections, were never seen crossing Tanglefoot barriers, so we assumed these barriers were highly effective at preventing woolly apple aphids from reaching tree canopies.

**Fig 4:**
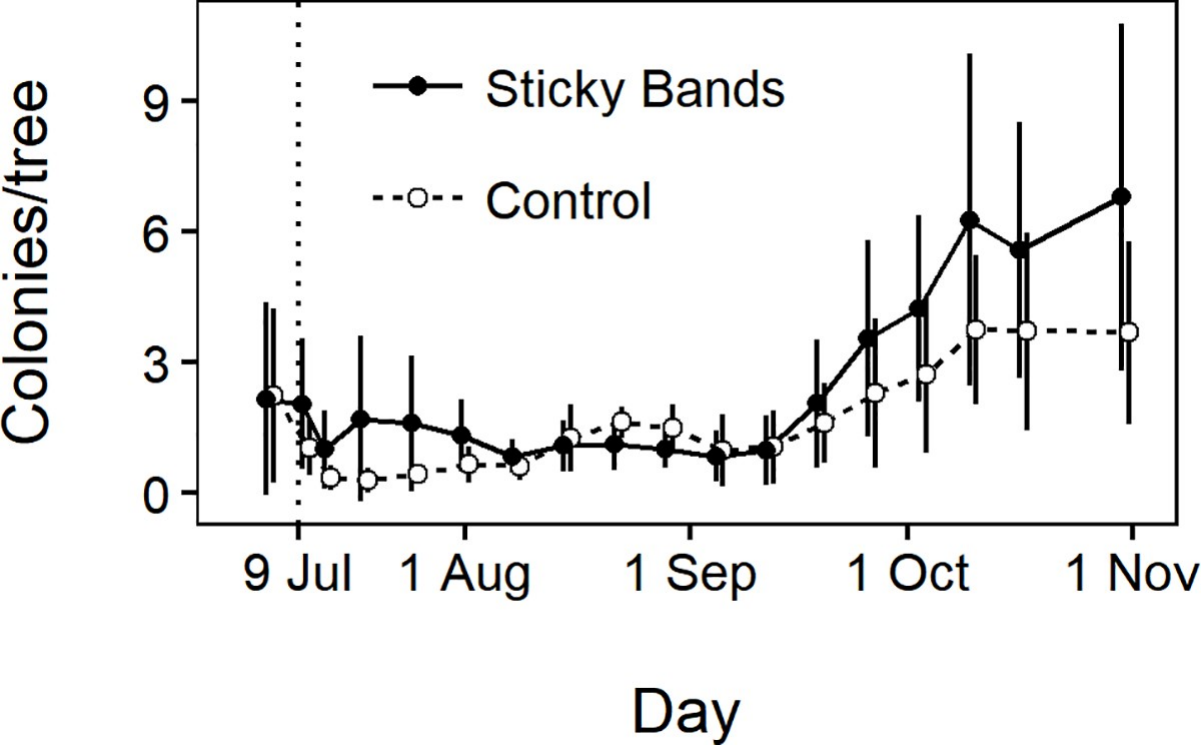
Mean aerial woolly apple aphid colonies per tree in the six control sections and six sticky band treatment sections ± 1 SD. The vertical dotted line shows when sticky bands were applied. Points are offset to avoid visual overlap of SD bars.

Woolly apple aphid aerial colonies were found across the entire observation period (Fig 4). Before the application of sticky bands, the number of aerial woolly apple aphid colonies was similar between the control (mean = 2.24 colonies per tree) and sticky band (mean = 2.16) treatments (*t* = -0.06, df = 10, *P* = 0.95) on 5 Jul. Following this, colony counts remained low, but control sections decreased to a minimum of 0.10 colonies per tree, while sticky band sections tended to have higher counts, never below 0.24 colonies per tree. Starting in mid-September, there was population growth in both treatments until November when cold weather reduced growth rates. Woolly apple aphid colony counts were not affected by sticky band treatment (*χ*^*2*^ = 1.6, *df* = 1, *P* = 0.21), but were affected by time (*χ*^*2*^ = 718, *df* = 17, *P* < 0.0001), and the time × treatment interaction (*χ*^*2*^ = -2.6, *df* = 17. *P* < 0.0001). The significant interaction reflects the greater increase in aerial woolly apple aphid colonies in sticky banded trees relative to control trees at the end of the season. Cumulative aerial colony-days per tree did not differ between control sections (mean = 207, SE = 39.5) and sticky band sections (mean = 308, SE = 69.7) (*t* = -1.38, *df* = 10, *P* = 0.20).

Woolly apple aphid nymphs with wing buds were not observed in colonies until 20 September (5 aphids out of 769 nymphs) and were found on all subsequent sampling days until 31 October (S2 Table). No winged adult was seen until 25 September (1 aphid out of 238 adults). Peak percentage of nymphs with wing buds (8.3%) occurred on 11 October, as did percentage of adults with wings (7.4%).

**Fig 5:**
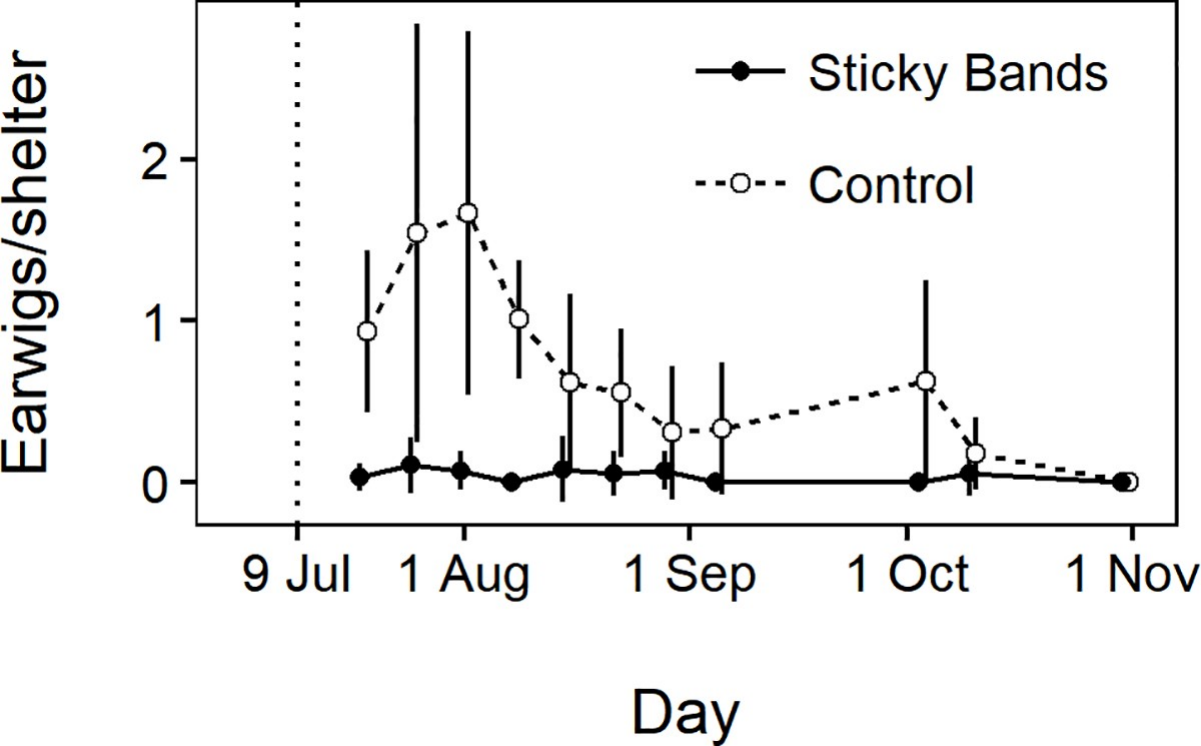
Mean earwig counts per shelter in the six control sections and six sticky band treatment sections ± 1 SD. The vertical dotted line shows when sticky bands were applied. Points are offset to avoid visual overlap of SD bars.

Earwigs were more abundant in control compared with sticky band sections (*χ*^*2*^ = 37, *df* = 1, *P* < 0.0001), and there was a significant effect of time (*χ*^*2*^ = 39, *P* < 0.0001), but not the time × treatment interaction (*χ*^*2*^ = -4.3, *df* = 9, *P* = 0.89) (Fig 5). Cumulative earwig-days per shelter were greater in control sections (mean = 61.8, SEM = 11.0) than in sticky band sections (mean = 3.1, SEM = 1.2) (*t* = 5.3, *df* = 10, *P* < 0.0001). Displacement of earwigs from shelters in tree canopies to the orchard floor during sampling may have contributed to the lower earwig counts in sticky band sections, as sampled earwigs in these sections would have greater difficulty returning to the canopies. However, earwig counts were already lower in sticky band sections than control sections at the first post-treatment observation (Fig 5), and no more than one earwig was ever found in any shelter in a sticky banded tree. Therefore, we believe bias introduced by our earwig sampling was small compared with the main exclusionary effect of the sticky bands on earwig abundance in the sticky banded trees.

### Greenhouse experiment

**Fig 6:**
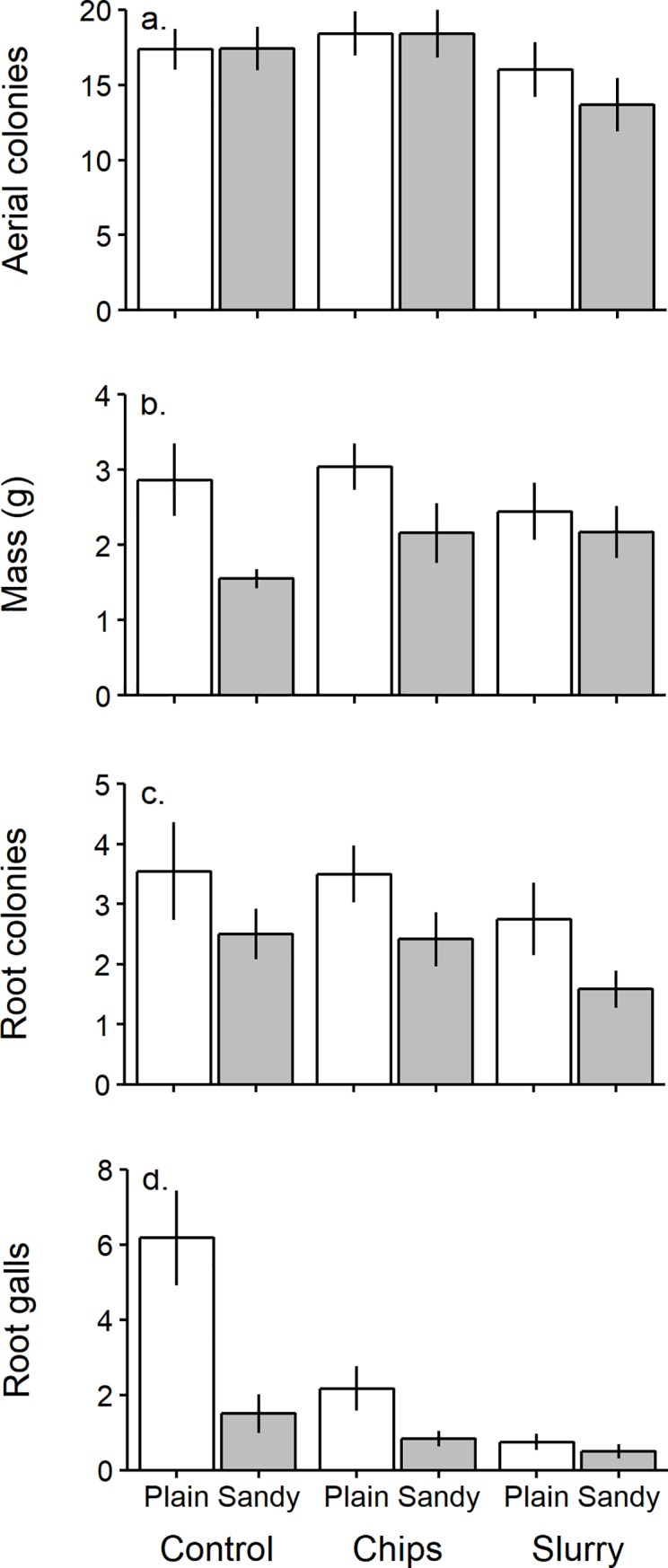
Measurements on greenhouse experiment trees. Shown are (A) number of aerial woolly apple aphid colonies, (B) root dry mass, (C) number of root woolly apple aphid colonies, and (D) number of root galls on potted apple trees. Bars show mean ± 1 SEM.

The number of aerial aphid colonies was marginally affected by mulch treatment (*F*_2,65_ = 2.7, *P* = 0.074), but was not affected by potting medium (*F*_1,65_ = 0.34, *P* = 0.56), or the mulch × potting medium interaction (*F*_2,65_ = 0.36, *P* = 0.69) (Fig 6A). Root dry mass was higher in plain compared with sandy media (*F*_1,65_ = 8.1, *P* = 0.006), but was not affected by mulch (*F*_2,65_ = 0.69, *P* = 0.51) or the potting media × mulch interaction (*F*_2,65_ = 1.1, *P* = 0.34) (Fig 6B). There were fewer root aphid colonies in sandy compared with plain media (*F*_1,65_ = 6.4, *P* = 0.013), but no effect of mulch treatments (*F*_2,65_ = 1.7, *P* = 0.20) or the potting media × mulch interaction (*F*_2,65_ = 0.01, *P* = 0.99) (Fig 6C). There were fewer root galls in sandy compared with plain media (*χ*^*2*^ = 38.4, *df* = 69, *P* < 0.001), and in wood chip or paper slurry mulch treatments (*χ*^*2*^ = 63.8, *df* = 67, *P* < 0.001), with no significant media × mulch interaction (*χ*^*2*^ = 3.2, *df* = 65, *P* = 0.20) (Fig 6D).

**Fig 7:**
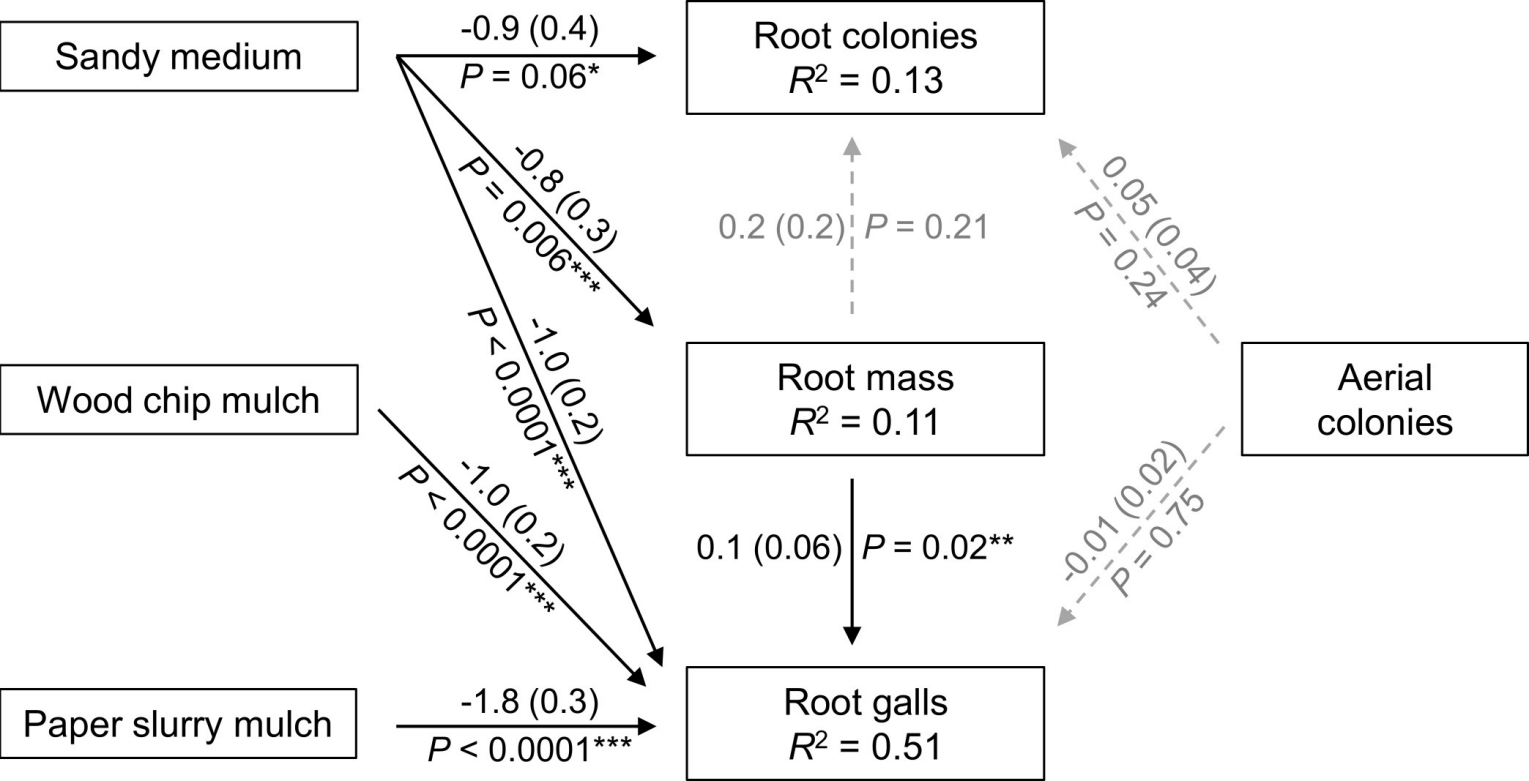
Path diagram from piecewise structural equation model of greenhouse tree data. Coefficient estimates are shown with standard error in parentheses and *P* values for each path. Non-significant paths (all *P* > 0.21) are shown in grey with dotted lines. Significant paths are shown in black with solid lines (*P <* 0.006 shown with three stars, *P* = 0.023 shown with two stars), and one marginally significant path (*P* = 0.057, shown with one star). *R*^2^ values correspond to component models.

The structural equation model (Fig 7) fit the greenhouse experiment data well (AIC = 33.6, Fisher’s *C* = 5.6, *df* = 8, *P* = 0.69). Sandy potting media and lower root weights both reduced gall counts. By lowering root weight, sandy media also indirectly lowered gall counts. Based on model coefficients, the direct effect of sandy media on gall counts was estimated 12.5 times greater than the indirect effect (calculated by multiplying the effect of sandy media on root mass with the effect of root mass on gall counts). Root galls were also significantly decreased by wood chip and paper slurry mulch treatments, whereas variation in aerial colony count did not affect occurrence of root galls. In addition, there was a marginal negative effect from sandy media on root colony counts.

## Discussion

Blocking movement of pests into available habitats, such as crop fields, can be an effective tactic to reduce pest abundance [1,39]. However, restricting movement could have little effect on a pest’s abundance if some individuals (such as winged adults) can bypass barriers, if there are existing local populations before barriers are applied, or if other population-regulating factors are more important [40]. In our study, earwig counts were lower on sticky-banded trees, possibly helping to explain why, rather than having fewer aboveground woolly apple aphid colonies, these trees had more woolly apple aphid colonies than control trees at some times. We also found that mulches partially restricted woolly apple aphid movement to roots in the greenhouse, and we would not expect mulches restrict to earwig movement into trees in the field. Use of mulches might thus by a practical way to improve woolly apple aphid management without interfering with biological control. However, our results suggest that restricting aphid movement may have weaker effects than biological control and other factors on aerial aphid populations.

Edaphic woolly apple aphids could be a source population in apple orchards sheltered from extreme temperatures and most insecticides. These populations may permit re-infestation of aerial populations [14,15,17], which often crash to undetectable levels only to reappear later [16]. However, our study and past studies have not found support for this hypothesis, as barriers on apple tree trunks have not consistently decreased aerial woolly apple aphid abundance [41,42]. Furthermore, in other studies, aerial abundance was not generally correlated to the timing of upward woolly apple aphid crawler movement [13,16,17] or to measures to edaphic abundance [20,31]. In these past studies, lateral aerial movement of crawling aphids between trees could have confounded interpretations, but we controlled for this by using spatially separated tree sections, with sticky barriers on trellising wires connecting adjacent sections. If upward movement is important in some situations, our observations might be explained by three hypotheses: (1) winged woolly apple aphids bypassed barriers and contributed to aerial population growth in both treatments, (2) other factors regulated woolly apple aphids in both treatments so movement made no considerable contributions, or (3) movement is only considerably important in re-establishing previously eradicated woolly apple aphid aerial populations but is not considerably important in increasing abundance of extant populations.

The hypothesis that winged woolly apple aphids can undermine physical barriers and cause aerial population growth [41,42] is not supported by our study because, similar to Beers et al. [16], winged aphids were not found until September. By this time, similar trends between treatments were already established in our study. Furthermore, winged woolly apple aphids have greatly reduced fecundity compared with wingless adults, and often produce sexual forms whose progeny can only grow on the overwintering host, American elm (*Ulmus americana* L.) [14,43–45]. The potential dispersal role of winged woolly apple aphids, which usually occur in autumn but can also occur in spring and summer in some parts of the world [44], remains unclear, especially in areas where American elm does not occur.

Low survival of upward moving crawlers may explain why restricting their movement did not reduce aerial woolly apple aphid abundance. Mols [46] estimated that in the field only 5% of woolly apple aphid crawlers (originating from aerial colonies) successfully founded new colonies, and Hoyt and Madsen [14] inferred that “a very small percentage” of woolly apple aphids emerging from the soil probably survive. In our study, increased predation by earwigs in control areas could have reduced population gains from upward-moving aphids compared with sticky banded trees, where earwigs were excluded from tree canopies. Although we did not monitor other natural enemies of woolly apple aphid, such as coccinellids, chrysopids, syrphids, and *Aphelinus mali* (Hymenoptera: Aphelinidae), these have winged adults and we assume would not have been excluded by sticky bands. However, it is possible that *A*. *mali* could be trapped and killed on sticky barriers [17], lowering their abundance. We had wanted to exclude effects of earwigs on woolly apple aphids by working in a location where earwig abundance was low, and we always found < 2 earwigs per shelter in control rows. Although this was low compared with estimates from other studies on the number of earwigs needed for effective woolly apple aphid control [33,47–49], correlational analyses [47,49] suggest that earwig counts of < 2 per shelter could decrease woolly apple aphid abundance compared with situations where no earwigs are found. In our study, even a small earwig population could have provided significant biological control if there were few alternative food sources other than woolly apple aphid colonies, but investigation of this possibility was beyond the scope of our study.

Aerial woolly apple aphid populations never completely disappeared at any point in our study, and it is possible that edaphic emigrants could help re-establish previously eradicated aerial woolly apple aphid populations. Therefore, longer-term studies assessing the effects of woolly apple aphid crawler movement on aerial population dynamics under different contexts could reveal situations where crawler movement is more important than it was in our study. However, because woolly apple aphids can successfully overwinter aboveground [16,17,32] and can often be found beneath bark (presumably sheltered somewhat from natural enemies and pesticides), it is not clear to what extent woolly apple aphid populations may ever completely disappear from canopies. The results of our field study suggest that aerial woolly apple aphid populations can rebound from low levels in the presence or absence of upward-moving crawlers.

In the greenhouse, we found fewer woolly apple aphid root galls on trees potted with sandy media, with wood chips, or with paper slurry mulch. According to our piecewise structural equation model (Fig 7), the number of root colonies did not differ with respect to these treatments, except for a marginal negative effect of sandy media (*P* = 0.06). The reduction in root galls without a reduction in woolly apple aphid root colonies could have occurred because root galls are reflection of the cumulative aphid abundance across time [19], whereas abundance can increase or decrease at different times. It is also possible that the treatments created an environment unsuitable for sustained feeding (and thus gall formation) or altered plant physiology so that gall formation was less possible. The lower root weight of trees grown in sandy media, and marginally lower number of aerial colonies on trees with paper slurry mulch, could have been an indication of tree stress or growth patterns associated physiological differences which might affect aphid feeding or gall formation.

Soil sand content and mulches could affect woolly apple aphid edaphic abundance in the field, but their management utility is currently unclear. Marcovitch [9] tested effects of soil texture on woolly apple aphid root colony abundance in the field and greenhouse and, similar to our greenhouse experiment, found that sandier soils had less root infestation. The apparent mechanism was that clay soils crack when dry, providing room for woolly apple aphids to crawl underground. Root populations of grape phylloxera (*Daktulosphaira vitifoliae* [Fitch]), a pest on grape roots, are thought to be similarly suppressed by sandy soil [10,50,51]. Our study suggests mulches could have a similar suppressive effect as sand. Paper slurry is not used commercially as mulch, but has been used experimentally [52]. Wood chips are used in some commercial apple orchards, and Brown and Tworkoski [53] found that a 6-cm layer of manure with wood chips slightly reduced the number of woolly apple aphids found in pitfall traps in apple orchards. Mulches in our study reduced aphid root galls in the greenhouse but did not completely prevent root infestation, so their benefits for woolly apple aphid edaphic management are questionable. It remains possible that mulches could reduce edaphic woolly apple aphid infestations over the long-term in the field, and could reduce edaphic population establishment in new orchards, but it is unlikely to be a strong control tactic.

We conclude that apple orchards on sandy soils or ones which use mulches are potentially less susceptible to woolly apple aphid root infestation, but aerial woolly apple aphid populations are typically not strongly affected by movement from edaphic populations. Recently released woolly apple aphid resistant rootstocks from the Geneva breeding program [23] are a promising tool for woolly apple aphid management, but our results suggest elimination of edaphic populations might not have a considerable influence on aerial population dynamics. Other studies have shown that woolly apple aphids can still be found in high numbers on susceptible scions grafted to the older resistant rootstocks MM106 and MM793 [47,48,54]. More generally, our study suggests that factors other than movement, such as biological control, might have more effect on woolly apple aphid population dynamics.

## Supporting information

S1 Table Raw data on aphid colony counts, earwig shelter counts, and woolly apple aphids on double-sided sticky tapes in the field experiment plot. (CSV)

S2 Table Raw data on woolly apple aphid colony monitoring for nymphs with wing buds and adults with wings at the field experiment plot. (CSV)

S3 Table Raw data on woolly apple aphid root and aerial colonies, root galls, and root dry weights in the greenhouse experiment. (CSV)
